# Myxedema Coma: Recognition of a Rare Endocrine Emergency

**DOI:** 10.7759/cureus.66053

**Published:** 2024-08-03

**Authors:** José Guilherme Assis, Anabela Santos

**Affiliations:** 1 Department of Medicine, Centro Hospitalar de Trás-os-Montes e Alto Douro, Vila Real, PRT; 2 Intensive Care Unit, Centro Hospitalar de Trás-os-Montes e Alto Douro, Vila Real, PRT

**Keywords:** endocrine emergency, side effects of amiodarone, sepsis, myxedema coma, hypothyroidism

## Abstract

An 82-year-old patient with multiple comorbidities presented to the emergency department with progressive dyspnea, orthopnea, and anorexia. Despite initial treatment for community-acquired pneumonia and decompensated heart failure, her condition deteriorated, manifesting as severe hypotension, bradycardia, and refractory hypothermia. A detailed medical history and extensive systematic investigation led to the documentation of hypothyroidism complicated by myxedema coma, in the context of chronic amiodarone use and precipitated by sepsis. Treatment with intravenous levothyroxine and glucocorticoids resulted in significant clinical improvement, leading to eventual hospital discharge. This case highlights the complexity and diagnostic challenges of myxedema coma, emphasizing the importance of early recognition, appropriate application of diagnostic scoring systems, and describing key aspects of the proper management of this rare endocrine emergency, whose symptoms and clinical signs are nonspecific.

## Introduction

Myxedema coma represents the most severe manifestation of hypothyroidism, constituting a critical, life-threatening endocrine emergency. This condition predominantly affects women and the elderly, owing to the elevated prevalence of hypothyroidism in these populations [1]. The risk of secondary hypothyroidism is further exacerbated by infection, various medications, chronic illnesses, trauma, hypothermia, and surgery [2]. Advanced age, hypotension or bradycardia at presentation, the requirement for mechanical ventilation, persistent hypothermia, sepsis, use of sedative medications, and decreased level of consciousness are substantial predictors of in-hospital mortality, which may reach 25-60% despite optimal therapeutic measures [1,3].

Diagnosis is based upon a confluence of clinical evaluation, encompassing signs of hypothyroidism, hypothermia, hyponatremia, hypercarbia, and hypoxemia, alongside laboratory assessment of thyroid-stimulating hormone levels [3,4]. While various diagnostic scores have been proposed, no universally accepted criteria have been established [2]. Treatment involves the administration of thyroid supplements and steroid replacement, coupled with supportive measures that include ventilation, management of hypotension, and correction of hypothermia [4].

This case report elucidates the diagnostic complexities and clinical management of an elderly woman with intricate comorbidities, who was ultimately diagnosed with myxedema coma in the context of sepsis and concurrent chronic amiodarone therapy.

## Case presentation

An 82-year-old female presented to the emergency department of a tertiary care hospital with progressive dyspnea, orthopnea, and anorexia. Previously autonomous and living alone, she had no documented close family connections. Her medical history included obesity, dyslipidemia, arterial hypertension, chronic kidney disease, chronic heart failure, atrial fibrillation, and osteoporosis. Her chronic medication regimen comprised candesartan 8 mg every 24 hours (q24h), amiodarone 200 mg q24h, apixaban 2.5 mg every 12 hours (q12h), furosemide 40 mg q24h, simvastatin 20 mg q24h, omeprazole 20 mg q24h, polystyrene calcium sulfate 20 g every 48 hours (q48h), allopurinol 150 mg q24h, alfacalcidol 0.5 µg q24h, mirtazapine 30 mg q24h, and alprazolam 1 mg q12h. Laboratory tests performed one month prior to admission indicated a decline in renal function, with serum creatinine levels increasing from 1.9 mg/dL to 2.6 mg/dL.

Upon physical examination, the patient exhibited drowsiness, a blood pressure of 125/74 mmHg, bradycardia (atrial fibrillation with a ventricular response of 50 bpm on ECG), a corporal temperature of 31°C, oxygen saturation of 90% on ambient air, bilateral pulmonary crackles on auscultation, and non-pitting peripheral edema. Arterial blood gas analysis revealed respiratory acidemia: pH = 7.26, partial pressure of carbon dioxide (PaCO2) = 70 mmHg, partial pressure of oxygen (PaO2) = 62 mmHg, bicarbonate (HCO3-) = 31.4 mmol/L, and lactate = 0.7 (results corrected for body temperature). Initial laboratory results are delineated in Table 1.

**Table 1:** Laboratory workup upon presentation.

Test	Result	Reference range
Hemoglobin	10.10 g/dL	12.0 – 16.0
Hematocrit	31.2%	37 – 49
Mean corpuscular volume	94.1 ft	87 – 103
White blood cell	12.50 x10^3^/uL	4.0 – 11.0
Neutrophils	83.9% (10.49 x10^3^/uL)	53.8 – 69.8
Lymphocytes	7.8% (0.98 x10^3^/uL)	25.3 – 47.3
Monocytes	7.7% (0.96 x10^3^/uL)	4.7 – 8.7
Eosinophils	0.6% (0.08 x10^3^/uL)	0.6 – 4.6
Basophils	0.0% (0.00 x10^3^/uL)	0.0 – 1.5
Platelets	184 x10^3^/uL	150 – 400
Glucose	212 mg/dL	82 – 115
Urea	158 mg/dL	<50
Creatinine	2.60 mg/dL	0.6 – 1.1
Sodium	133 mEq/L	135 – 147
Potassium	4.4 mEq/L	3.7 – 5.1
Aspartate aminotransferase	40.0 U/L	<35
Alanine transaminase	25 U/L	<33
Alkaline phosphatase	139 U/L	25 – 105
γ-glutamyl transferase	24 U/L	7 – 12
Lactate dehydrogenase	257 U/L	135 – 214
Total bilirubin	0.2 mg/dL	<1.2
C-reactive protein	22.2 mg/dL	<0.5
Pro B-type natriuretic peptide	880.0 pg/mL	<120

**Figure 1:**
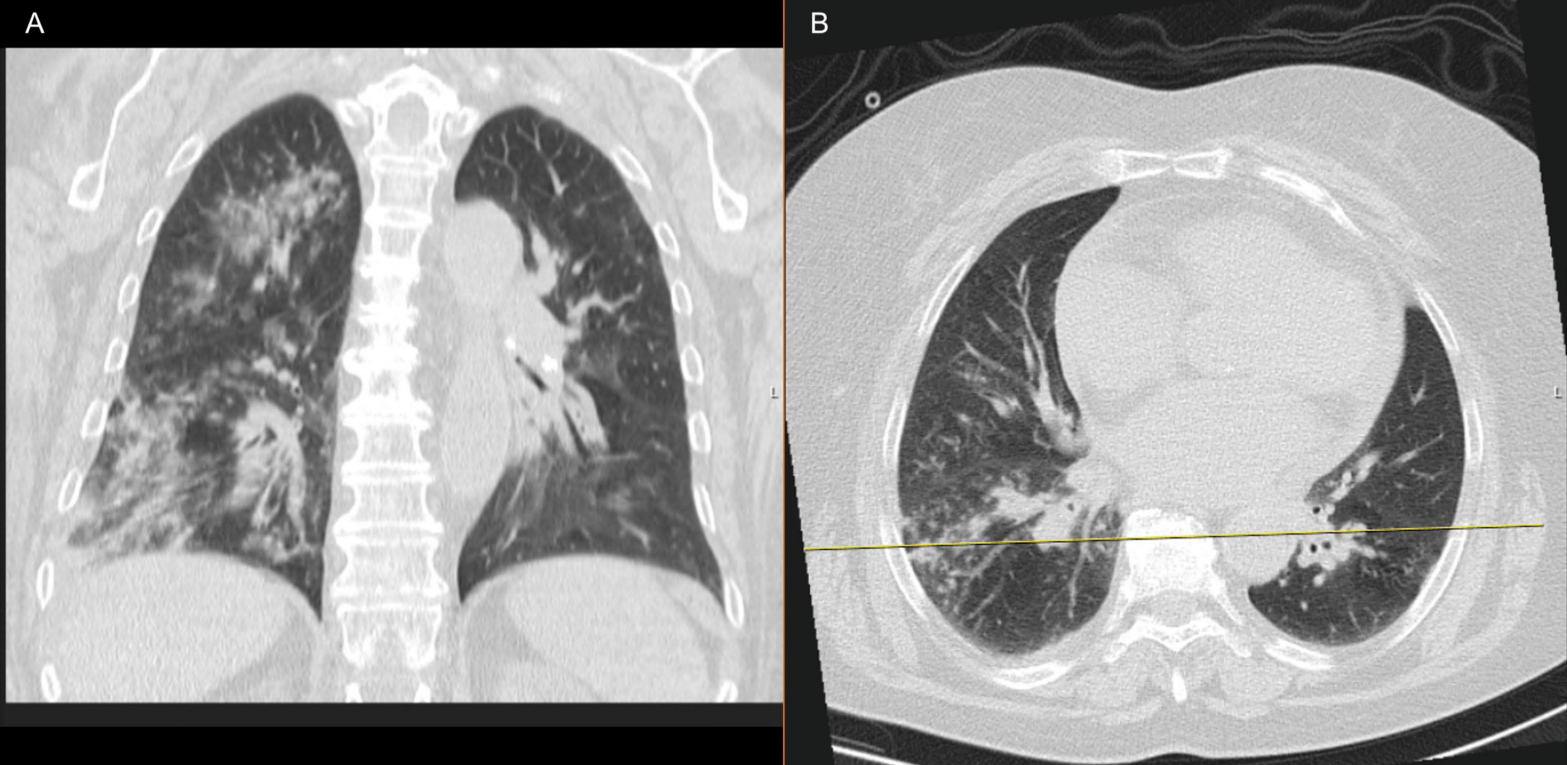
Computed tomography of the chest: coronal view (panel A) and axial view (panel B). Consolidation in the right upper, middle, and lower lobes is associated with mucoid impaction in the small airways of the latter. Subpleural opacities in the posterior fields of both lungs. Small bilateral pleural and pericardial effusions.

Urine sampling was performed to rule out infection. Thoracic computed tomography revealed an asymmetric infiltrate, predominantly in the right upper lobe and right pulmonary base, as shown in Figure 1. Pertinent samples were collected for microbiological testing. Treatment was initiated with diuretics, bronchodilators, hydrocortisone 200 mg IV to address transient bronchospasm, and empirical antibiotic therapy (amoxicillin/clavulanate 1200 mg IV every eight hours (q8h), combined with azithromycin 500 mg IV q24h). Non-invasive ventilatory (NIV) support was started due to the unresponsiveness of respiratory acidosis to initial interventions.

Despite these measures, the patient’s condition deteriorated, developing lethargy, severe bradycardia, hypotension refractory to fluid resuscitation, and persistent hypothermia. Consequently, she was transferred to the intensive care unit, where vasopressor support with norepinephrine, isoprenaline perfusion, and active external rewarming were initiated. She had an initial Acute Physiology and Chronic Health Evaluation II (APACHE II) score of 28 and a Sequential Organ Failure Assessment (SOFA) score of 12. Cardiac point-of-care ultrasound revealed preserved biventricular function (eyeball), a small pericardial effusion, and a dilated inferior vena cava without inspiratory collapse.

On day one, escalating vasopressor demands were observed despite normal lactate levels and sustained urinary output. Although hypercapnia improved, hypoxemia aggravated. The patient continued to experience hypothermia and altered mental status (Glasgow Coma Scale score of 14). Isoprenaline was effective in controlling heart rate. Altered coagulation was identified, but the patient was under treatment with a factor Xa inhibitor (international normalized ratio = 1.25, normal range: <1.2; activated partial thromboplastin time = 57.1 s, normal range: 24-35). Given the patient's evolving clinical condition, further investigation was warranted.

Investigation

Blood cultures were pending results. Respiratory virus screening, including SARS-CoV-2, and urinary antigen tests for *Streptococcus pneumoniae* and *Legionella pneumophila* returned negative. Procalcitonin levels were measured at 0.2 ng/mL (low risk when <0.5 ng/mL). Cardiac biomarkers, including cardiac troponin T (0.030 ng/mL, normal reference <0.05 ng/mL), were assessed. D-dimer assay and computed tomography pulmonary angiography were deferred as pulmonary embolism was considered unlikely. Toxicology screening was omitted given the insufficient supporting history or clinical evidence.

Endocrine tests were prompted as part of the diagnostic pathway. Adrenocorticotropic hormone (ACTH) levels were measured and found to be 11.1 pg/mL (normal range: <63 pg/mL), with serum cortisol levels not assessed due to previous steroid administration. Thyroid function tests showed a markedly elevated thyroid-stimulating hormone (TSH) level of 172 µIU/mL (normal range: 0.27 - 4.20 mIU/L) and a severely reduced free thyroxine (FT4) level of 2.4 pmol/L (normal range: 10.3 - 22.2 pmol/L).

Differential diagnosis

The diagnostic approach encompasses a broad spectrum of conditions given the complex clinical presentation of this elderly patient. Initial considerations included sepsis and pneumonia, supported by observed respiratory symptoms, physical examination findings, elevated inflammatory markers on initial laboratory tests, and imaging results. However, the lack of response to antibiotic therapy and persistent hypotension despite adequate fluid resuscitation prompted consideration of alternative or concurrent pathologies.

Acute heart failure emerged as a significant differential diagnosis, underpinned by the patient's medical history of chronic heart failure, hypertension, and atrial fibrillation, alongside clinical features suggestive of hypervolemia with recent declining renal function, and elevated pro-B-type natriuretic peptide. Despite treatment attempts with standard therapies such as diuretics and non-invasive ventilation, there was insufficient improvement, leading to progressive respiratory decline, persistent hypotension, and deteriorating gas exchange. Acute coronary syndrome was also considered due to cardiovascular risk factors and initial hypotension and bradycardia, but excluded based on normal troponin levels and absence of ischemic changes on ECG. Disseminated intravascular coagulation and pulmonary embolism were excluded due to coagulation results and low clinical suspicion (Wells' criteria for pulmonary embolism ≤ 4 points). Medical voluntary intoxication was considered improbable due to the lack of history suggesting voluntary medical intoxication and its unlikely explanatory role for the observed clinical manifestations.

Ultimately, consideration turned to endocrine causes due to the patient’s persistent hypothermia, bradycardia, altered mental status, and lack of clinical response, and upon reviewing the medical history, highlighting the chronic prescription of amiodarone 200 mg q24h (previously discontinued since admission). This assessment was reinforced by the patient’s multisystem involvement, encompassing cardiovascular abnormalities, neurological manifestations, and hypoventilation, confirmed by profound thyroid function abnormalities. These factors collectively led to the established diagnosis of hypothyroidism complicated by myxedematous coma precipitated by sepsis and medication. This finding was confirmed using a diagnostic scoring system (Table 2).

**Table 2:** Application of diagnostic scoring system for myxedema coma. >60 points: highly suggestive/diagnostic of myxedema coma; 25-59 points: supportive of a diagnosis of myxedema coma; <25 points: myxedema coma unlikely. The diagnostic scoring system was adapted from [5].

Variable	Applied factor	Points
Thermoregulatory dysfunction (temperature, °C)	<32	20
Central nervous system effects	Somnolent/lethargic	10
Gastrointestinal findings	Absent	0
Precipitating event	Present	10
Cardiovascular dysfunction		
Bradycardia	<40	30
Other ECG changes	Absent	0
Pericardial/pleural effusions	Present	10
Pulmonary edema	Present	15
Cardiomegaly	Absent	0
Hypotension	Present	20
Metabolic disturbances		
Hyponatremia	Present	10
Hypoglycemia	Present	10
Hypoxemia	Present	10
Hypercarbia	Present	10
Decrease in glomerular filtration rate	Present	10
Total score		165

Treatment and follow-up

Prompt recognition and initiation of appropriate treatment were critical in effectively managing this life-threatening condition. The treatment regimen included continued supportive measures, glucocorticoids (hydrocortisone 100 mg IV q8h), and levothyroxine 200 µg IV q24h - liothyronine was unavailable. Concurrent pneumonia was managed with empirical antibiotic therapy despite the absence of an isolated pathogen.

The patient exhibited significant clinical improvement, with progressive normalization of neurological status despite occasional agitation. Temperature and electrolyte levels returned to normal, enabling the successful discontinuation of non-invasive ventilation, vasopressors, and isoprenaline. On day three, levothyroxine administration was transitioned to the oral route. Peripheral edema markedly improved, and thyroid function normalized (Table 3); triiodothyronine (T3) levels were not assessed due to the absence of liothyronine. On day six, the patient was transferred to the general ward. Following rehabilitation and oxygen weaning, she was discharged from the hospital on day 21. She resumed her daily activities, with follow-up appointments scheduled for medical reevaluation. On day 22 post discharge (day 43 after initial hospital admission), her ambulatory evaluation documented the stabilization of thyroid function under treatment with levothyroxine 125 µg orally q24h.

**Table 3:** Timeline of thyroid function tests, clinical signs, and symptoms. TSH: thyroid-stimulating hormone.

Test	Day 1	Day 2	Day 3	Day 4	Day 13	Day 21	Hospital discharge	Day 43
TSH (0.27 – 4.20 mlU/L)	127.00	80.20	51.00	46.00	36.87	16.31		4.41
Free T4 (10.3 – 22.2 pmol/L)	2.40	5.30	6.70	8.20	14.43	18.70		
Signs and symptoms								
Bradycardia	+							
Hypotension	+	+	+					
Hypoxemia	+	+	+	+	+	+		
Hypercapnia	+							
Pulmonary edema	+	+	+	+	+			
Lethargy	+							
Confusion		+	+	+				
Anasarca	+	+	+	+				
Hypothermia	+							
Hyponatremia	+							
Hypoglycemia	+	+	+					

## Discussion

Encephalopathy in the elderly encompasses a heterogeneous array of etiological factors, including disturbances in thyroid function. Myxedema coma constitutes a severe complication of decompensated hypothyroidism, frequently triggered by a multitude of underlying precipitating factors, and is characterized by a non-specific clinical presentation [4].

As depicted in this case, this condition predominantly afflicts elderly women, with infection identified as the leading precipitant. The chronic administration of amiodarone warrants particular scrutiny as a potential concurrent contributory factor. Amiodarone impedes the peripheral conversion of thyroxine (T4) to T3 and obstructs the cellular uptake of T4, leading to elevated serum T4 levels, reduced serum and intrapituitary T3 concentrations, and subsequently elevated serum TSH levels, as evidenced in this case. Although amiodarone-induced myxedema coma is exceedingly rare - documented in fewer than 20 instances since 1971 - it typically arises between three months and two years following the initiation of therapy, particularly at doses exceeding 200 mg/day [2]. The patient's protracted amiodarone regimen, extending over a decade, may either diminish its import as a putative etiological factor or warrant the contemplation of a possible protracted temporal correlation - an aspect that remains plausible given the extant limited data. Additional relevant factors in this case include a history of chronic heart failure and the use of sedative medications and diuretics, which may exacerbate or obscure the diagnosis [1].

The pathophysiology of myxedema coma involves reduced intracellular T3 due to hypothyroidism, leading to hypothermia and diminished cardiac function. Respiratory failure may arise from hypoxia insensitivity, hypercapnia, respiratory muscle dysfunction, and airway obstruction. Altered vascular permeability can result in pericardial and/or pulmonary effusions and anasarca, with hyponatremia secondary to renal dysfunction and excess vasopressin. Cardiac depression and hypotension may precipitate cardiogenic shock, which is often refractory to vasopressors without appropriate treatment. Additionally, hypoglycemia and central nervous system depression may lead to seizures and altered consciousness, while gastrointestinal dysfunction, alopecia, bladder dystonia, distension, and anemia further complicate the clinical picture [4]. A diagnostic scoring approach for myxedema coma was employed to corroborate clinical suspicion and facilitate earlier recognition and intervention [5].

According to the American Thyroid Association task force, the initial management of myxedema coma should include intravenous levothyroxine, with the optional addition of liothyronine [6]. Recent evidence suggests that oral levothyroxine might serve as an effective alternative when intravenous administration is not feasible [7]. Glucocorticoid therapy is recommended for all patients, given the potential coexistence of hypoadrenalism with hypothyroidism, as treating hypothyroidism alone may precipitate adrenal insufficiency [8]. Despite initially high APACHE II and SOFA scores upon ICU admission, strong predictors of adverse outcomes [9], the patient exhibited a favorable clinical trajectory.

## Conclusions

A detailed medical history, consideration of potential precipitating factors, a high index of suspicion, a comprehensive diagnostic approach, including thyroid function tests, and an appropriate diagnostic scoring system are essential for diagnosing myxedema coma, a rare endocrine emergency with nonspecific symptoms and signs. Initial treatment includes supportive measures, intravenous hydrocortisone, and intravenous levothyroxine (oral route may be considered). Early detection and timely management can lead to significant clinical improvement and favorable patient outcomes despite severe initial presentations.
